# Rooted in Excellence: The Life and Work of Dr. John I. Ingle

**DOI:** 10.7759/cureus.76910

**Published:** 2025-01-04

**Authors:** Sumeet Agarwal, Laresh N Mistry, Himmat Jaiswal, Shreyas Neelkanthan, Shantanu Deshpande, Saudamini More, Hrashikesh Vaidya

**Affiliations:** 1 Department of Prosthodontics, Bharati Vidyapeeth (Deemed to be University) Dental College and Hospital, Navi Mumbai, IND; 2 Department of Pediatric and Preventive Dentistry, Bharati Vidyapeeth (Deemed to be University) Dental College and Hospital, Navi Mumbai, IND; 3 Department of Conservative Dentistry and Endodontics, Bharati Vidyapeeth (Deemed to be University) Dental College and Hospital, Navi Mumbai, IND; 4 Department of Public Health Dentistry, Bharati Vidyapeeth (Deemed to be University) Dental College and Hospital, Navi Mumbai, IND

**Keywords:** biography, clinical dentistry, dr. john i. ingle, endodontics, root canal therapy

## Abstract

Dr. John I. Ingle significantly influenced the field of dentistry, particularly in endodontics, where his work has reshaped clinical practices and educational frameworks. Renowned for his pivotal role in modernizing root canal therapy, Ingle’s efforts have greatly enriched the global understanding of dental science. His landmark textbook, "Ingle’s Endodontics", first published in 1965, remains a cornerstone in the discipline, offering comprehensive guidance on endodontic principles and practices. Additionally, his leadership in establishing advanced academic programs, notably at the University of Washington, has played a transformative role in training and mentoring successive generations of dental practitioners. This analysis explores Ingle’s early life, academic journey, and enduring contributions to the development and dissemination of endodontic knowledge. Through his innovations in both clinical techniques and educational standards, Ingle has left an indelible mark on the evolution of dentistry.

## Introduction and background

Dr. John I. Ingle was born on June 6, 1919, in Colfax, Washington, marking the beginning of a life dedicated to significant contributions to science and healthcare. His early years were shaped by a rigorous educational foundation, which eventually led him to pursue a Doctor of Dental Surgery (DDS) degree at Northwestern University. From a young age, Ingle demonstrated a keen interest in healthcare and education, coupled with an exceptional academic aptitude. These qualities laid the groundwork for his remarkable career in dentistry, particularly in the specialized field of endodontics, where his innovations and leadership would leave a lasting impact on the profession [1].

Dr. John I. Ingle's formative years in a rural setting instilled a strong work ethic and a dedication to public service, both of which profoundly influenced his professional trajectory. Early exposure to demanding labor and altruistic pursuits shaped his values and decision-making throughout his career. After completing his undergraduate education, he pursued advanced studies in dentistry at Northwestern University, where he demonstrated exceptional skills in both clinical practice and academic endeavors.

Ingle engaged in advanced academic pursuits and earned a Master of Science in Dentistry (MSD), signifying the initiation of an extensive career characterized by significant contributions to the fields of dental education and practice. His engagement with the emerging endodontics field ignited during his postgraduate studies, establishing a foundation for a professional trajectory that would result in significant innovations in root canal therapy and dental education [1].

## Review

Military service and its impact

Following his graduation, during World War II, Dr. Ingle served in the U.S. Army Dental Corps. This period was pivotal, as it allowed him to refine his clinical skills in high-pressure environments and address complex oral health challenges faced by military personnel. His service instilled in him a profound appreciation for the role of dentistry in improving quality of life, a theme that would underpin his future endeavors. The experience also reinforced his commitment to evidence-based practice and the importance of comprehensive dental care [2].

Early contributions to dentistry

Following the attainment of his Doctor of Dental Surgery degree, Dr. Ingle embarked on a professional journey that would extend over several decades and significantly transform dental practices on a global scale. During the late 1940s and early 1950s, the field of endodontics exhibited a notable lack of development, with a significant number of practitioners lacking formal training in the techniques associated with root canal therapy [3]. In response to the necessity for standardized methodologies and enhanced results, Dr. Ingle directed his efforts toward the progression of endodontics, a specialized area within dentistry that examines and addresses the dental pulp and the tissues encircling the root of a tooth.

In 1955, Dr. Ingle disseminated his inaugural significant article, in which he introduced standardized methodologies for root canal therapy, emphasizing the critical nature of canal length assessment and the appropriate shaping of the canal to achieve favorable results [3]. The emphasis on meticulousness and precision in root canal therapy established foundational principles that significantly influenced the discipline.

Dr. Ingle is recognized for his significant contributions to the formal establishment of endodontics as a distinct dental specialty. In 1959, he established the Graduate Endodontics Program at the University of Washington, which was among the earliest programs of its type in the United States. His objective was to educate clinicians who possessed not only exceptional practical skills but also engaged in academic and scientific inquiry within the discipline. The program emerged as a paradigm for endodontic education on a global scale and has been instrumental in the development of numerous prominent figures in the field of dentistry, such as Dr. Mahmoud Torabinejad, who is recognized for his pioneering work in the formulation of mineral trioxide aggregate, an important material utilized in endodontic procedures.

Landmark publication: Endodontics

The discipline of endodontics has significantly benefited from the foundational contributions of Dr. Ingle, particularly through his influential textbook, "Endodontics", which was initially released in 1965 [4]. This textbook is extensively recognized as a highly authoritative resource within the discipline. Ingle's text provided an extensive analysis of the endodontics domain, presenting a novel classification framework for root canal treatment failures and promoting the necessity of scientific precision in clinical methodologies. His book played a crucial role in shaping the education and practices of numerous dental students and practitioners over the years [1].

One of the significant advancements introduced by Dr. Ingle was the standardized methodology for the preparation and obturation of root canals. Prior to his contributions, the practice of root canal therapy exhibited significant variability, resulting in inconsistent patient outcomes. Dr. Ingle's research highlighted the necessity for rigorous standards in quantifying the length of the root canal, alongside the critical significance of effective cleaning, shaping, and sealing procedures [5].

Contributions to academic and clinical endodontics

Dr. Ingle's impact reached far beyond the confines of his textbook. In the year 1960, he assumed the role of chairman for the Department of Endodontics at the University of Southern California (USC), maintaining this position until 1970. Throughout his tenure, he significantly advanced USC's status as a leading institution for endodontic research and training [6]. The leadership exhibited led to the establishment of one of the pioneering formal endodontic programs, integrating comprehensive clinical training with advanced research methodologies.

Ingle's methodology regarding endodontic education underscored the importance of evidence-based practices and the ongoing enhancement of techniques. Under his leadership, the curriculum at USC focused on the empirical and applied dimensions of root canal therapy, ensuring that students were adequately prepared to navigate the intricacies of endodontic treatment.

Throughout this timeframe, Dr. Ingle initiated significant contributions toward the formation of professional organizations aimed at promoting the advancement of endodontics. He served as a founding member of the American Association of Endodontists, which was established in 1943 [7], and significantly influenced its developmental trajectory.

He proposed the establishment of certification standards for endodontists and supported the acknowledgment of endodontics as a distinct specialty within the field of dentistry. The culmination of his advocacy efforts resulted in the formal acknowledgment of endodontics as a recognized specialty by the American Dental Association in the year 1963 [8].

Standardization of endodontic techniques

Dr. John Ingle made a notable contribution to the field of dentistry through his research focused on the standardization of endodontic techniques. During the 1960s, there existed a notable lack of standardization in the methodologies employed for root canal treatments, which resulted in a significant disparity in clinical outcomes. In response, Dr. Ingle formulated a comprehensive set of guidelines aimed at standardizing the procedure of root canal therapy. His research encompassed suggestions for quantifying canal length, the application of suitable instruments, and the closure of root canals [9].

The guidelines presented in later editions of his textbook established a fundamental basis for contemporary endodontic practice. Dr. Ingle's emphasis on precision, particularly regarding canal shaping and obturation, has consistently been a fundamental principle of endodontic therapy. The findings of his research contributed significantly to the understanding of the necessity of preserving the integrity of the periapical tissues throughout the process of root canal therapy [10]. The emphasis on tissue preservation has significantly transformed the field and enhanced patient outcomes.

Advances in endodontic instrumentation

Dr. Ingle played a crucial role in the enhancement of the instruments utilized in endodontic therapy. The investigation into root canal instruments conducted during the late 1960s and early 1970s resulted in the advancement of tools that enhance the efficiency and efficacy of the cleaning and shaping processes of root canals. One of his significant contributions was the development of the standardized K-file, an instrument utilized for the shaping of the root canal space [11]. This instrument, in conjunction with other advancements in endodontic tools, has markedly enhanced the precision and efficacy of root canal treatments. 

Dr. Ingle collaborated extensively with manufacturers to enhance the design of endodontic instruments, thereby ensuring their safety and efficacy in clinical applications. The innovations he introduced in this domain have resulted in a significant and enduring influence, with numerous instruments utilized in contemporary endodontics deriving from his foundational designs [12].

Clinical outcomes and research

Throughout his career, Dr. Ingle engaged in comprehensive investigations regarding the results of endodontic therapies. During the late 1970s, he initiated multiple longitudinal studies that monitored the efficacy of root canal treatments over durations ranging from 5 to 10 years. The studies, published in prominent dental journals, offered significant evidence that substantiated the effectiveness of contemporary endodontic techniques [13]. The findings of his investigation indicated that, when executed correctly, root canal therapy has the potential to maintain teeth for extended periods, thereby contesting the widely accepted belief at the time that extraction represented the optimal approach for compromised teeth [14].

Dr. Ingle’s research elucidated the variables that led to failures in endodontic procedures. The findings indicated that insufficient cleaning and shaping of canals, along with inadequate sealing, were principal factors contributing to the failure of the treatment [15]. The results of this study underscored the significance of following standardized methodologies and facilitated the broad implementation of his techniques.

Advocacy for evidence-based practice

Dr. Ingle was a staunch advocate for evidence-based practice, emphasizing the importance of research in driving clinical decision-making. He believed that dentistry, like all branches of medicine, should be grounded in rigorous scientific inquiry. To this end, he encouraged collaboration between researchers and clinicians, fostering a culture of innovation and critical thinking within the dental community.

His commitment to evidence-based practice is reflected in his numerous publications, which address a wide range of topics, from the etiology of endodontic infections to the long-term outcomes of root canal therapy. Through his work, Dr. Ingle underscored the importance of integrating scientific knowledge with clinical expertise to achieve optimal patient outcomes.

Impact on global dentistry

Dr. Ingle's influence extended far beyond the United States, shaping dental practices and education worldwide. He was a sought-after lecturer, sharing his expertise at conferences and academic institutions across the globe. His contributions to the development of standardized protocols and training programs have elevated the quality of endodontic care internationally.

Ingle’s leadership roles in professional organizations further amplified his impact. He served as a mentor to countless dental professionals, inspiring them to uphold the highest standards of care and to approach their work with integrity and compassion. His emphasis on lifelong learning and professional development has left a lasting impression on the global dental community.

Legacy and impact on modern dentistry

Dr. Ingle’s contributions to the discipline of dentistry, especially within the specialized area of endodontics, have resulted in significant and enduring effects. The sixth edition of the textbook, "Endodontics", remains a pivotal resource for dental students and practitioners across the globe [16]. The standardization of endodontic techniques he developed has been integrated into dental education globally, thereby facilitating the advancement of future dental practitioners through his innovative contributions.

Alongside his scholarly contributions, Dr. Ingle served as a mentor to numerous dental professionals. His dedication to education and his focus on methodological precision have impacted not only endodontics but also the wider discipline of dentistry. His research has significantly contributed to the enhancement of care standards within dental practices worldwide, and his impact is evident in the elevated success rates observed in contemporary endodontic treatments [17].

**Figure 1:**
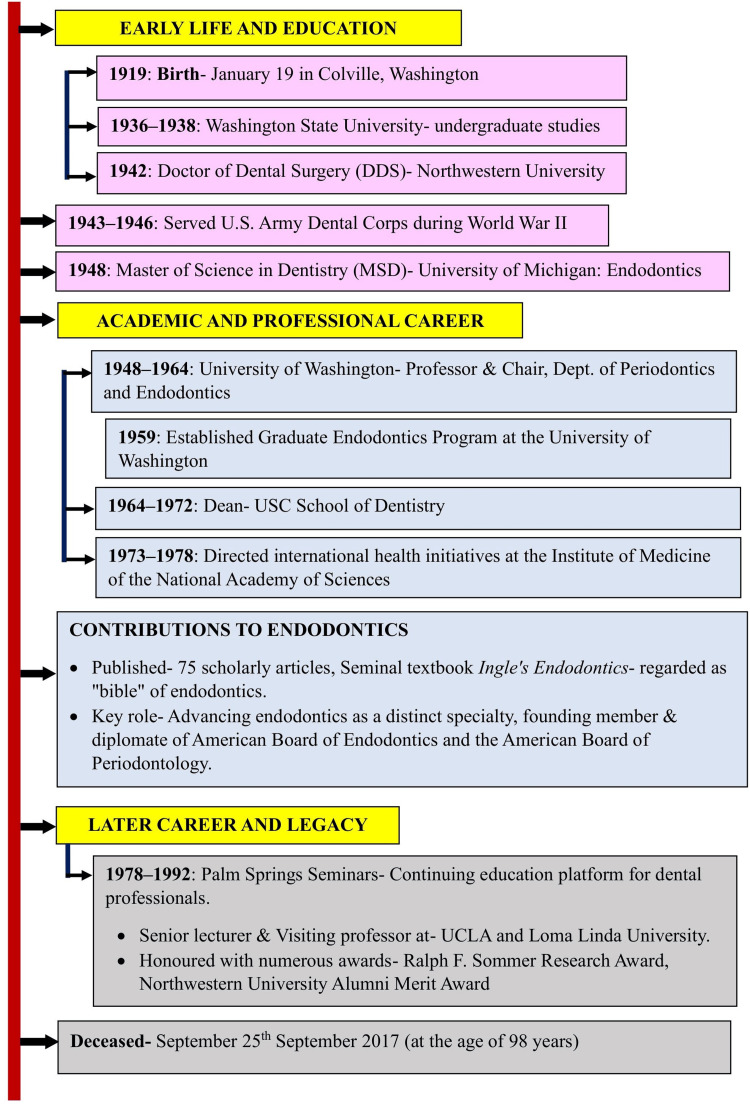
Timeline of Ingle's life journey Image credits: Dr. Shreyas Neelkanthan, Dr. Laresh N. Mistry

The timeline of Ingle's entire life journey is summarized (Figure 1). The legacy of Dr. Ingle is prominently reflected in the multitude of awards and honors conferred upon him during his professional career. In 1982, he received the Edgar D. Coolidge Award, the highest accolade bestowed by the American Association of Endodontists [2]. Furthermore, he demonstrated significant productivity as an author, contributing more than 100 articles to peer-reviewed journals throughout his professional tenure [18].

## Conclusions

The contributions of Dr. John I. Ingle in dentistry, especially within the domain of endodontics, are of significant magnitude. The standardization of root canal therapy, advancements in dental education, and enhancements in clinical outcomes resulting from his work have produced significant and enduring effects. The textbook "Endodontics" persists as a foundational element in dental education, while the innovations introduced in endodontic techniques are integral to contemporary practice. Dr. Ingle’s contributions exemplify a commitment to excellence, unwavering dedication, and an insatiable quest for knowledge, significantly influencing the discipline of dentistry for future generations.
